# Application of Infrared Pyrolysis and Chemical Post-Activation in the Conversion of Polyethylene Terephthalate Waste into Porous Carbons for Water Purification

**DOI:** 10.3390/polym16070891

**Published:** 2024-03-24

**Authors:** Mikhail Efimov, Andrey Vasilev, Dmitriy Muratov, Alexander Panin, Maria Malozovskaya, Galina Karpacheva

**Affiliations:** 1 A.V. Topchiev Institute of Petrochemical Synthesis RAS, Leninskiy Prospekt 29, 119991 Moscow, Russiagpk@ips.ac.ru (G.K.); 2 Department of Functional Nanosystems and High-Temperature Materials, National University of Science and Technology “MISiS”, Leninskiy Prospekt 4, 119049 Moscow, Russia; 3 Department of Electronics Materials Technology, National University of Science and Technology “MISiS”, Leninskiy Prospekt 4, 119049 Moscow, Russia; 4 National Research Center “Kurchatov Institute”, Academic Kurchatov Sq. 1, 123182 Moscow, Russia

**Keywords:** polyethylene terephthalate, infrared heating, pyrolysis, activated carbon, adsorbent

## Abstract

In this study, we compared the conversion of polyethylene terephthalate (PET) into porous carbons for water purification using pyrolysis and post-activation with KOH. Pyrolysis was conducted at 400–850 °C, followed by KOH activation at 850 °C for samples pyrolyzed at 400, 650, and 850 °C. Both pyrolyzed and post-activated carbons showed high specific surface areas, up to 504.2 and 617.7 m^2^ g^−1^, respectively. As the pyrolysis temperature increases, the crystallite size of the graphite phase rises simultaneously with a decrease in specific surface area. This phenomenon significantly influences the final specific surface area values of the activated samples. Despite their relatively high specific surface areas, pyrolyzed PET-derived carbons prove unsuitable as adsorbents for purifying aqueous media from methylene blue dye. A sample pyrolyzed at 650 °C, with a surface area of 504.2 m^2^ g^−1^, exhibited a maximum adsorption value of only 20.4 mg g^−1^. We propose that the pyrolyzed samples have a surface coating of amorphous carbon poor in oxygen groups, impeding the diffusion of dye molecules. Conversely, post-activated samples emerge as promising adsorbents, exhibiting a maximum adsorption capacity of up to 127.7 mg g^−1^. This suggests their potential for efficient dye removal in water purification applications.

## 1. Introduction

Polymers—familiar to everyone as household plastics in food and beverage packaging, kitchenware, interior decorations, and furniture; elements of clothing, such as synthetic fibers, shoes, and accessories; and components of household appliances, like casings and structural parts—are integral constituents of contemporary life with their wide range of applications in day-to-day living and various industrial sectors. These materials, due to their ease of production, cost-effectiveness, durability, chemical resistance, and excellent formability, have found widespread acceptance. Today, a huge number of synthetic polymers are being produced around the world to meet these varied needs [1].

Polyethylene terephthalate (PET), a synthetic polymer extensively employed in the food industry, is recognized for its chemical resistance and safe interaction with food and beverages, ensuring no harmful contaminants are transferred when used for packaging. Additionally, its transparency, plasticity, and low carbon dioxide permeability enhance its desirability. These remarkable properties make PET one of the leaders in the production of household plastics. However, as the annual consumption of this polymer increases, the waste produced rises proportionally, escalating the pressure on the environment [2]. Although PET poses no direct hazard to human health, the mounting volume of PET waste, combined with its resistance to natural degradation, underscores the necessity for efficient recycling strategies. The escalating volume of PET waste directly contributes to the increasing generation of microplastics [3]. These are micron-sized polymer particles that detach from the parent solid plastic material, infiltrating the water bodies of rivers, lakes, seas and oceans, as well as our soils, creating a discreet but serious environmental risk [4]. In addition, discarded packaging and other components of plastic waste can ensnare, suffocate or injure mammals, birds or fish that are incapable of freeing themselves.

Several approaches to PET waste recycling are known and these can generally be categorized into mechanical and chemical methods [5,6]. An example of a mechanical method is the re-extrusion of the polymer to create products with similar properties. However, this re-extrusion process often results in reduced mechanical strength of the polymer, which limits its utility, especially in applications within the food industry. This limitation is further magnified by the challenges associated with effectively sorting and purifying plastic waste prior to processing.

An alternative method for PET recycling involves chemolysis processes, including hydrolysis, glycolysis, methanolysis, and aminolysis [7,8,9]. These processes promote depolymerization, transforming polymer molecules into monomers or oligomers that are suitable for use as raw materials in the production of new products [10,11]. However, this technology is hampered by certain economic factors, such as the requirement for high-cost equipment capable of withstanding high pressures and the necessity for strong acids and alkalis.

There is a growing interest in obtaining valuable liquid and gaseous products, including hydrogen, from PET [12,13,14]. This includes thermal decomposition of the polymer in an inert atmosphere with the assistance of a catalyst, resulting in a variety of products such as liquid hydrocarbons, ethylene, propylene, wax, mono- and dioxide carbon, hydrogen, and solid carbon residue. PET pyrolysis can be considered as a dual simultaneous process: the cleavage of polymer chains yielding volatile compounds and the condensation of aromatic rings producing a carbon residue. The application of the latter, however, often tends to be overshadowed and does not receive the attention it deserves. This is partially attributed to the carbon residue’s structural and functional characteristics, which are often poorly suited to any role. For instance, according to the N_2_ adsorption procedure the specific surface area does not exceed 150 m^2^ g^−1^ after pyrolysis conducted at 950 °C [15]. A well-established method for the chemical activation of carbon materials exists, which involves the high-temperature heating of the carbon material in the presence of an activating agent, such as alkali, acid or metal salts [16].

PET-derived activated carbon material shows promise in various applications. It can act as a material for energy storage—particularly as an electrode material in supercapacitors [17] and serve as a support for metal nanoparticles, which are used as catalysts in a wide array of chemical reactions [18,19]. Additionally, PET-derived porous carbon is studied as an adsorbent for the purification of liquid and gaseous mediums from pollutants [20,21,22]. Porous carbons, including PET-derived carbons, are proposed to be used to purify water from dyes. Methylene blue (MB) dye, a commonly employed model pollutant in research, serves to elucidate the adsorption characteristics and mechanisms of carbons. Employing PET as a carbonaceous precursor offers two distinct advantages: its cost-effectiveness, resulting from the large quantity of its waste accumulated worldwide, and its relatively consistent composition and structure. This consistency is particularly advantageous when compared to sources like biomass, which typically exhibit more variability.

In this paper, we propose two approaches for PET utilization. The first approach concerns the direct pyrolysis of the polymer via infrared (IR) heating, leading to the production of porous carbon without the need for employing activating agents. The second strategy involves a conventional two-stage chemical activation process, consisting of PET carbonization followed by high-temperature treatment in the presence of potassium hydroxide. A notable difference between our study and existing research is the application of IR heating, which significantly reduces the energy and time costs associated with the PET conversion process. In addition, we demonstrate for the first time the influence of the preliminary pyrolysis temperature on the structure and properties of the PET-derived activated carbon. The structural and adsorption characteristics of the resulting porous carbons are then compared.

## 2. Materials and Methods

### 2.1. Materials and Porous Carbon Preparation

Light blue transparent plastic bottles (from still water drink) sliced into 1 cm^2^ pieces were used as a PET source. We synthesized two sets of samples. The first set included PET samples that underwent pyrolysis at the range of 400–850 °C, including 400, 650, and 850 °C. Pyrolysis of PET was performed in a laboratory IR heating furnace [23] in a nitrogen atmosphere at a heating rate of 50 °C min^−1^ with exposure period of 2 min. The carbon materials produced within pyrolyzed set are labeled as PET-T, where T represents the pyrolysis temperature. 

The second set involves the post-activated carbon samples. This set consisted of samples pyrolyzed at 400, 600, and 850 °C that were then post-activated with KOH, all at the same temperature of 850 °C. These temperatures were chosen to study the effect of pretreatment temperature on the efficiency of KOH activation. For this, PET-T carbon powders were impregnated with an aqueous solution of potassium hydroxide (Fisher Chemical, Pittsburg, PA, USA, pure grade), sonicated using a 22 kHz ultrasound (Melfiz Ultrasound, Moscow, Russia) for 2 min and then kept for 24 h. The molar ratio of the carbon component in the PET-T samples to alkali (C to KOH) was set at 3 to 1. The carbon content was established using CHNS analysis. Then, powder was dried at 120 °C until constant weight followed by IR heat treatment at 850 °C in a nitrogen atmosphere at a heating rate of 50 °C min^−1^. Afterward, the resultant porous carbon materials were thoroughly washed to remove alkali, using distilled water until a neutral pH was achieved and then dried at 120 °C until a consistent weight was obtained. The samples produced by this method are denoted as PET-T-a, where *T* represents the pretreatment temperature.

### 2.2. Porous Carbon Characterization

The quantitative carbon, hydrogen, and nitrogen contents in the examined samples were established using a Flash 2000 CHNS analyzer (Thermo Fisher Scientific, Waltham, MA, USA). Surface area characteristics were assessed by nitrogen adsorption at −196 °C using a Nova 4200e apparatus (Quantachrome Instruments, Boynton Beach, FL, USA). The pore size distribution was determined employing the quenched solid density functional theory (QSDFT) model, facilitated by the Quantachrome NovaWin 11.06 software. The FTIR spectra of porous carbon materials were captured using a Bruker IFS-66v/s FTIR spectrometer (Bruker, Karlsruhe, Germany) in 3 cm^−1^ resolution and a 600–4000 cm^−1^ registration range. For this, KBr pellets were used. 

We obtained Raman spectra on a Senterra II spectrometer (Bruker, Billerica, MA, USA), utilizing a wavelength of 532 nm and power of 0.25 mW. We investigated the phase composition and structure of the obtained carbon materials by analyzing diffractograms captured on an X-ray diffractometer Diffray-401 (Scientific Instruments, St. Petersburg, Russia) at room temperature using Cr-Kα radiation (wavelength 0.22909 nm). Scanning electron microscopy (SEM) of the carbon powders was conducted on a Vega3 SB microscope (TESCAN, Brno, Czech Republic). We performed thermogravimetric (TG) analysis using a thermal analyzer, SDT Q-600 (TA Instruments, New Castle, DE, USA), in the temperature range of 25–1000 °C within an argon atmosphere at a heating rate of 10 °C min^−1^.

### 2.3. Adsorption Study

To investigate adsorption properties, the dependency of adsorption values on the contact time of the prepared sample with the methylene blue (LenReactiv, St. Petersburg, Russia, 99%) solution was examined. Each adsorption test involved introducing 0.05 g of the adsorbent into 50 mL of an aqueous MB solution, with concentrations ranging from 25 to 300 mg L^−1^. The solution’s pH was left unadjusted, approximately at pH 6.5. The resulting suspension was stirred by a shaker at 160 rpm at room temperature for varying time intervals, up to 240 min. Equilibrium adsorption tests were conducted for 4 h to ensure equilibrium was attained.

UV spectroscopy was employed to determine MB concentration in the water solution. The UV-vis spectrophotometer (Shimadzu UV-1700, Kyoto, Japan) measured the intensity of the peak at 664 nm, and these data were utilized to calculate the residual MB content applying a calibration curve. Adsorption tests were performed in duplicate, and the reported adsorption properties represent average values.

The time and equilibrium adsorption capacities of the prepared sample, denoted as *q_t_* and *q_e_* (in mg g^−1^), were determined using Equations (1) and (2):

(1) $$q_{t}=\frac{(C_{0}-C_{t})V}{m}$$

(2) $$q_{e}=\frac{(C_{0}-C_{e})V}{m}$$

where *C*_0_, *C_t_*, and *C_e_* (mg L^−1^) are initial, time, and equilibrium concentrations of MB, respectively; *V* (L) is the volume of the MB solution; and *m* (g) is the weight of the sample. 

Two equilibrium models, namely Langmuir and Freundlich, were used to fit experimental data. The pseudo-first-order (PFO), pseudo-second-order (PSO), and Elovich equations were used to study the kinetics of adsorption reactions. Corresponding equations are given in Supplementary Materials. 

The kinetic and isotherm model parameters were determined by nonlinear regressions using the software OriginPro 2017 (OriginLab, Northampton, MA, USA).

## 3. Results

### 3.1. Structural Characteristics of Pyrolyzed and Post-Activated PET Samples

In an effort to transform PET into a porous carbon material suitable for use as an adsorbent in water purification, carbon materials have been prepared via pyrolysis in the temperature range of 400–850 °C. In addition, we have demonstrated the potential for enhancing porosity characteristics and surface chemistry through the KOH-activation of the obtained carbon materials. The choice of the minimum processing temperature is caused by the unique thermal decomposition characteristics of PET. This polymer is well known for its good thermal resistance, with a melting onset at 260 °C and boiling initiation at 350 °C [24]. 

**Figure 1:**
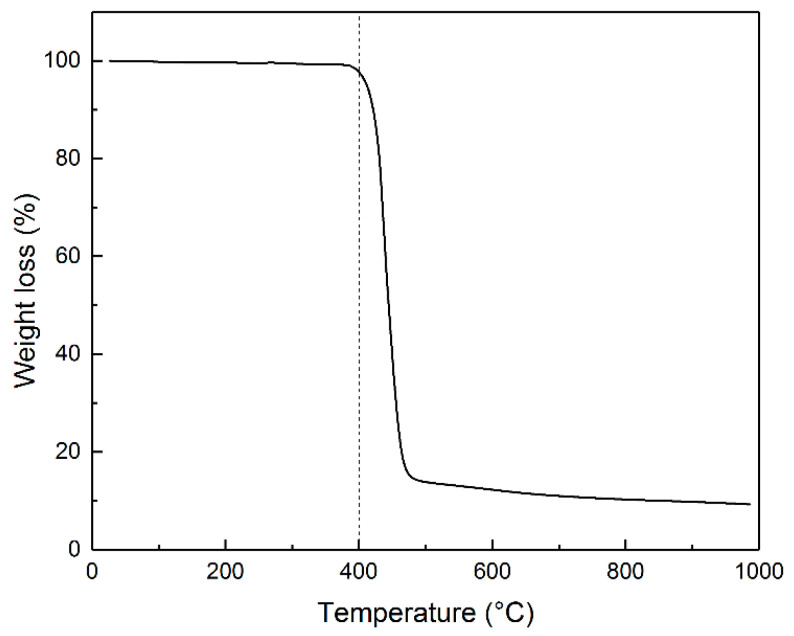
TG curve illustrating the change in PET weight when heated in an argon atmosphere at a heating rate of 10 °C min^−1^.

After melting and subsequent cooling, PET forms a dense, solid mass that resists grinding, posing challenges for uniform impregnation with an alkaline solution. The TG curve in Figure 1 indicates the polymer decomposition starting around 390 °C. At 400 °C, the polymer transforms into a black, brittle substance that is easily crushable and visually resembles carbon powder (Figure S1).

The use of IR heating procedure can significantly save pyrolysis, carbonization and activation time. Thus, conventional heating is usually carried out at a rate of 5–10 deg min^−1^ and requires 0.5–2 h of holding time [16]. The laboratory IR heating furnace allows heating at a rate of 60 deg min^−1^. The exposure time is only 2 min. Cooling of the quartz reactor is also relatively short-term. The pyrolysis stage with the parameters specified in the Experimental Part requires up to 5800 kJ, which is about 2 times less than the energy consumption of pyrolysis in a conventional furnace [25].

**Figure 2:**
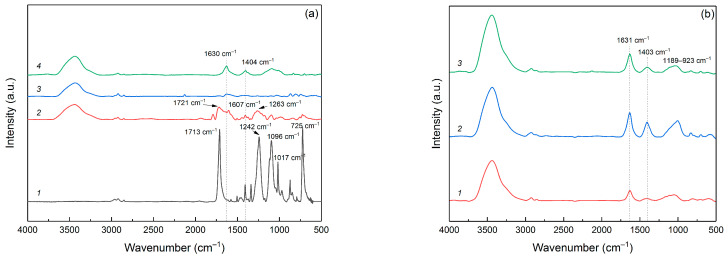
IR spectra of (**a**) initial PET (1), pyrolyzed samples PET-400 (2), PET-650 (3), PET-850 (4) and (**b**) activated samples PET-400-a (1), PET-650-a (2), and PET-850-a (3).

In Figure 2, a comparative analysis is depicted through IR spectra, covering the original PET bottle sample, pyrolyzed PET at varying temperatures and activated samples. As illustrated in Figure 2a, the initial PET spectrum exhibits several distinct bands. The bands observed at 2967, 2922, and 2855 cm^−1^ correspond to the stretching vibrations of the =C–H and C–H bonds within the ethylene component of PET. The prominent band at 1713 cm^−1^ associates with the ether C=O bonds. Another strong band at 1242 cm^−1^ represents the C–O–C bonds, which form the linkage between the terephthate and ethylene segments. The band at 1096 cm^−1^ corresponds to the ester O–C–C bonds associated with the methylene group of ethylene glycol [26,27], whereas the band at 1017 cm^−1^ refers to the ester C–O bonds [28]. The 725 cm^−1^ band could to be ascribed to rocking vibrations in the CH_2_-CH_2_ within aliphatic chain [29].

The spectrum of the PET-400 significantly changes compared to the spectrum of the initial polymer. Thus, the band from the C=O bond in the ether group of the polymer shifts to 1721 cm^−1^, which indicates the cleavage of the bond between the terephthalate and the aliphatic chain of ethylene, which provides the formation of a complete carboxyl bond with the formation of acid on one side and vinyl ether on the other, according to the scheme of temperature transformation of PET proposed in [30,31]. This transformation is also indicated by a marked decrease in the intensity of bands at 1096, 1017 and 1242 cm^−1^, which can be attributed to ester C-O bonds, including those binding the terephthalate part to ethylene glycol. In addition, a very intense 1263 cm^−1^ band appears in the PET-400 sample spectrum, clearly indicating the formation of benzoic acid [32]. The presence of a band at 1607 cm^−1^, associated with C=C in the aromatic ring, is quite noteworthy and may suggest the initiation of the decarboxylation process of the terephthalate part of the material via benzoic acid degradation. Despite the considerable transformations of the polymer after pyrolysis at 400 °C, the spectrum of this sample indicates that a significant portion of the initial PET monomer units is preserved. 

The spectrum of the PET-650 sample reflects substantial changes compared to the initial polymer and the features linking this spectrum to the previous ones are barely distinguishable. For instance, the band associated with the carbonyl C=O bonds, situated around 1720 cm^−1^, is barely detectable. The intense band at 1630 cm^−1^, the trace of which can also be seen in the spectrum of the PET-400 sample, refers to the aryl group. This indicates the connection of aromatic rings and the beginning of formation of graphite layers. The weak band at 1263 cm^−1^ indicates a minor benzoic acid content and completion of the decarboxylation process. Furthermore, the absence of the 725 cm^−1^ band indicates the total disintegration of the initial compound of terephthalic acid and ethylene glycol.

Pyrolysis of PET at 850 °C leads to the formation of a graphite-like structure, which is characterized by the presence of surface oxygen groups. Accordingly, the 1630 cm^−1^ band exhibits a markedly increased intensity compared to the preceding spectra, which indicates the formation of graphite planes. The spectrum also displays broad bands at 1404 cm^−1^ and within the 1225–930 cm^−1^ range, corresponding to C-O and C-O-C bonds [33], indicating functional oxygen groups on the surface of the carbon structure. It is noteworthy that the higher pyrolysis temperature provided a more oxidized carbon structure of the resulting material. At the same time, the PET-650 sample contains very little oxygen according to FTIR spectrum analysis.

Comparison of the IR spectra of post-activated carbon materials based on pyrolyzed PET, as presented in Figure 2b, reveals that varying pretreatment temperatures do not significantly alter the surface’s chemical bonds. The band at 1631 cm^−1^ indicates C=C bonds in aromatic rings and indicates the formation of graphite layers. At the same time, the intensity of this band in the PET-400-a sample is the lowest, which indirectly suggests a lower degree of graphitization for the sample. However, it should be noted that post-activation leads to the formation of oxygen groups on the surface of the carbon material. This is especially noticeable if we compare FTIR spectra of PET-650 and PET-650-a samples. The band at 1403 cm^−1^ refers to the C-O and C-O-C bonds. The broad band 1189-923 cm^−1^ also indicates the presence of oxygen groups on the surface of the C-O and C-O-C carbon material and is typical of activated carbon materials. Such materials, in addition to possessing a high specific surface area, have a surface saturated with functional oxygen groups.

All the post-activated samples prepared display the bands at 2961, 2925, and 2852 cm^−1^, indicative of C–H bond vibrations. In addition, a broad and intense band within the 3000–3660 cm^−1^ range is observable, revealing the presence of OH–groups, which are likely a result of water adsorption in the KBr pellets.

**Figure 3:**
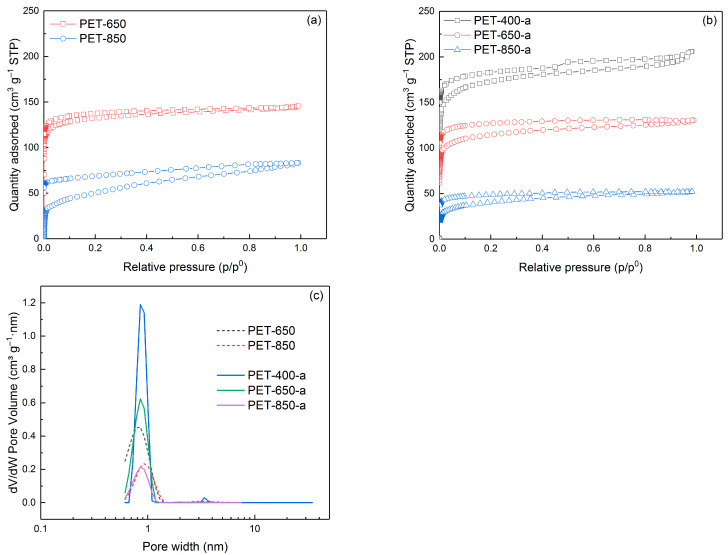
N_2_ adsorption/desorption isotherms of (**a**) pyrolyzed and (**b**) activated PET samples as well as (**c**) pore size distribution.

The study of porosity of the obtained pyrolyzed and activated samples by nitrogen adsorption/desorption method revealed some regularities. Figure 3 illustrates nitrogen adsorption/desorption isotherms, which highlight the differences in porosity of the obtained samples.

As seen, the isotherms of all samples belong to Type I isotherms, which are characterized by a horizontal plateau and describe microporous solids. The exception is the isotherm of the PET-400-a sample, which is a combination of type I and type II. Nevertheless, hysteresis loops present in most of the isotherms indicate the presence of mesopores.

The pore size distribution is presented in Figure 3c. As can be seen from the figure, all samples have pore sizes predominantly less than 1 nm. The PET-400-a sample has some considerable amount of mesopores (>2 nm), which is explained by the parallel processes of carbonization of the polymer and its activation in the presence of KOH. Thus, oxidation of the carbon structure prevents it from forming extended graphite-like sites, remaining defective.

Table 1 compiles data on the porosity characteristics of the carbon materials obtained. It has been found that an escalation in pyrolysis temperature corresponds to a decrease in specific surface area, dropping from 511.7 to 221.0 m^2^ g^−1^. The specific surface area of the PET-650 sample, selected for further investigation, measured as 504.2 m^2^ g^−1^. No data are available for the PET-400 sample due to its incomplete polymer degradation, as confirmed by FTIR spectrum analysis (Figure 2a). Consequently, attempting to degas the sample to obtain a nitrogen adsorption/desorption isotherm at lower temperatures (<400 °C) leads to partial decomposition, resulting in an incomplete isotherm and introducing a certain level of error in specific surface area measurement. To our best knowledge, very few works are devoted to direct pyrolysis of PET to obtain porous carbons [34,35,36]. We show for the first time that it is possible to pyrolyze PET to obtain porous carbon.

The data clearly indicate that increasing the initial pyrolysis temperature significantly influences the final porosity of the activated samples. After 400 °C, the processes of polymer decomposition begin dramatically with the release of gaseous products, the main of which are CO and CO_2_ [37]. When the temperature is relatively low (650 °C, for instance), it is sufficient for decomposition and release of gaseous products, which ensures the formation of a porous structure. However, a further increase in pyrolysis temperature involves a competing process—the ordering of the carbon structure (building up graphite sites). Thus, the pores are healed.

Consequently, as the pretreatment temperature rises, there is a notable decrease in specific surface area—from 617.7 m^2^ g^−1^ for the PET-400-a sample to 166.0 m^2^ g^−1^ for the PET-850-a sample. The elevated pyrolysis temperature promotes the development of a sturdier structure characterized by a higher degree of graphitization. Graphite, being less susceptible to oxidation during alkali activation, accounts for the observed decline in specific surface area with increasing pyrolysis temperature. In addition, a noteworthy observation is that the activation of the pyrolyzed samples surprisingly did not enhance the specific surface area values; instead, there was a slight decrease.

Table 1 also contains the results of the elemental CHN analysis. Quite expectedly, no nitrogen was found in any sample. The oxygen content, which was calculated in terms of the content of other elements, strongly depends on the pyrolysis temperature.

The PET-400 sample exhibits 22 wt% of oxygen according to the analysis, attributed to the incomplete decomposition of the polymer at this temperature (the original PET contains 33.3 wt% of oxygen). Subsequent to polymer decomposition, at 550 °C, the sample registers a reduced oxygen content of 3.3 wt%. However, increasing the pyrolysis temperature to 850 °C boosts the oxygen content to 13.8 wt%. Since pyrolysis occurs in an inert gas flow, it can be assumed that the pyrolyzed samples possess numerous open bonds, and oxygen is bound to the surface of the carbon material after the sample is removed from the reactor. Samples activated at the same temperature but with different preliminary pyrolysis temperatures do not differ much in elemental composition. The oxygen content is in the range of 10.3–12.7 wt% for all samples. Although here it should be noted that there is a persistent trend of increasing oxygen content with increasing preliminary pyrolysis temperature.

**Figure 4:**
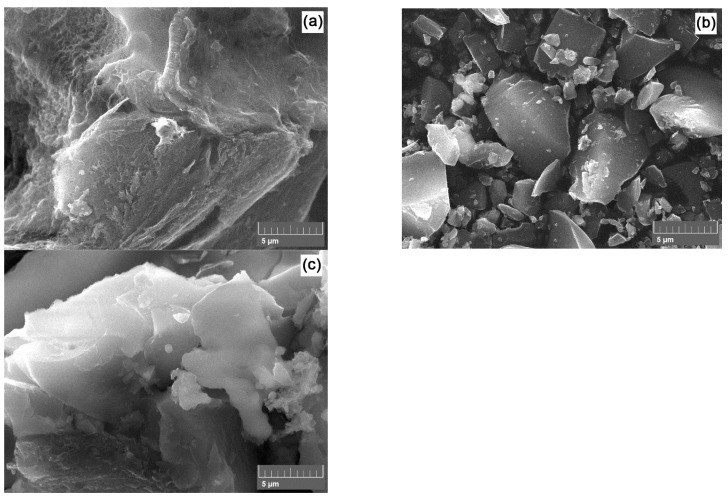
SEM micrographs of PET-400-a (**a**), PET-650-a (**b**), and PET-850-a (**c**) samples.

Figure 4 presents SEM micrographs of the post-activated carbon materials. 

Visually, the morphology of these powders varies according to their specific surface area. Thus, the PET 400-a sample, characterized by the largest specific surface area, has a relative relief surface with the presence of thin carbon layers.

Additionally, open cavities and the spongy surface morphology of the carbon material are visible. The morphology of samples PET-650-a and PET-850-a is quite different and represents shard-like particles. The particle surface of the carbon material appears denser, indicating less interaction between the activating agent and carbon. It is worth noting that micropores, which contribute significantly to the total specific surface area, are not distinguishable in the SEM photographs.

**Figure 5:**
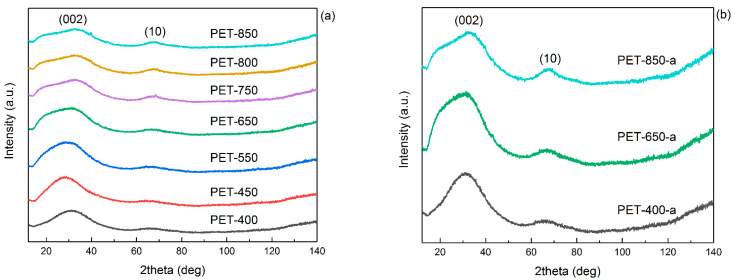
XRD patterns of (**a**) pyrolyzed and (**b**) post-activated PET samples.

The evolution of the crystal structure of the resulting carbon-containing material can be traced by XRD patterns, which are presented in Figure 5.

Figure 5a shows the comparison of XRD patterns of pyrolyzed PET at different temperatures. It can be seen that the structure of the carbonaceous material changes with increasing pyrolysis temperature. Thus, the carbonaceous material obtained at 400–450 °C is quite different from the higher temperature samples. As can be seen from the diffractograms, all samples have a diffuse peak from the (002) plane of graphite. At the same time, higher pyrolysis temperature leads to the appearance of asymmetry at the peak on the small angle side, which may indicate a more oxidized structure. This is indirectly confirmed by the analysis of FTIR spectra. At the same time, the increase of pyrolysis temperature leads to the appearance and more pronounced expression of the asymmetric (10) peak, the intensity of which may indicate the degree of graphitization of the material. The crystallite size of L_a10_ was calculated using the Scherrer equation (Equation (3)) with a Warren correction factor equal to 1.84 (Table 2) [38].

(3) $$L_{a10}=\frac{\lambda K}{\beta \cos\theta }$$

where *λ* (nm) is the wavelength (0.22909 nm for Cr-Kα radiation), *θ* (radians)—the Bragg angle, *K* is a constant (Warren factor) and *β* is the full width at half maximum of the intrinsic X-ray line broadening (radians).

It showed that the crystallites do not enlarge up to 550 °C, keeping a size of 1.7 nm. Starting from 650 °C, an increase in crystallite size was observed up to 2.4 nm at 850 °C. Thus, an increase in temperature leads to an increment in the graphitic plane size, which confirms an increase in the degree of graphitization of the material. The appearance of XRD patterns is rather typical for amorphous carbon materials.

Notably, the asymmetric peak (10) becomes narrower and more pronounced with the rise in preliminary pyrolysis temperature. The calculation of crystallite size indicates a slight increase with the elevation of pyrolysis temperature (Table 2). For instance, PET-400-a is characterized by a crystallite size of 2.6 nm, while PET-850-a displays a size of 2.9 nm. Thus, the repeated heating of the sample during post-activation results in an increased crystallite size compared to the pyrolyzed samples. This increase becomes more noticeable, particularly when the activation temperature at 850 °C exceeds the pyrolysis temperature by a greater margin.

Another technique that provides detailed insights into the structure of carbon materials is Raman spectroscopy. Typically, amorphous carbon materials exhibit a spectrum featuring two broad bands, known as the G- and D-bands. The G-band, positioned around ~1580 cm^−1^, characterizes the stretching of sp^2^ bonds in the graphite basal plane. The D-band (~1340 cm^−1^) arises from the breathing mode of carbon atoms in the ring, indicating disorder in the graphite material.

The interpretation of carbon material spectra has a longstanding history dating back to the 1970s. In general, spectrum analysis is condensed by determining the ratio of D and G bands. Tuinstra and Koenig first claimed that the crystallite size (the reference value obtained through calculation using the Sherrer equation from XRD analysis data) is inversely proportional to the ratio of *I_D_*/*I_G_* band intensities [40]. Ferrari and Robertson then stated that the *I_D_*/*I_G_* ratio is directly proportional to *L_a_^*2*^* if the crystallite size is less than 2 nm [41]. Cancado et al. proposed an universal formula (Equation (4)) for calculating the crystallite size using the ratio of the areas of D- and G-peaks [39].

(4) $$L_{a}=(2.4\times 10^{-10})\lambda^{4}{\left(\frac{A_{D}}{A_{G}}\right)}^{-1}$$

where *λ* (nm) is the wavelength and *A_D_* and *A_G_* are the areas of the D- and G-peaks, respectively.

To achieve this goal, it is essential to identify the peak pattern, which, in turn, requires deconvoluting the entire spectrum. Various approaches exist for decomposing the spectrum, involving fitting 2-5 peaks [42,43,44]. However, when they are applied to calculate crystallite size, they can give an error due to poorly chosen deconvolution parameters, for example, if different peak models (Gaussian, Lorentzian, Voigt) are used for the compared peaks of different spectra. Mallet-Ladeira et al. [45] applied an approach in which the D1 and D2 peaks have the same center on the Raman shift scale, which is explained by double resonance [46]. In previous work, we have shown that for some materials, in particular amorphous activated carbons, it is not always possible to use three-peak deconvolution [47]. This approach yields a non-optimal sum spectrum in the “saddle” region between the D- and G-peaks. In this study, we also used a combined approach of fitting four Gaussians to the sample spectrum. In this case, the centers of the D1 and D2 peaks have the same value.

**Figure 6:**
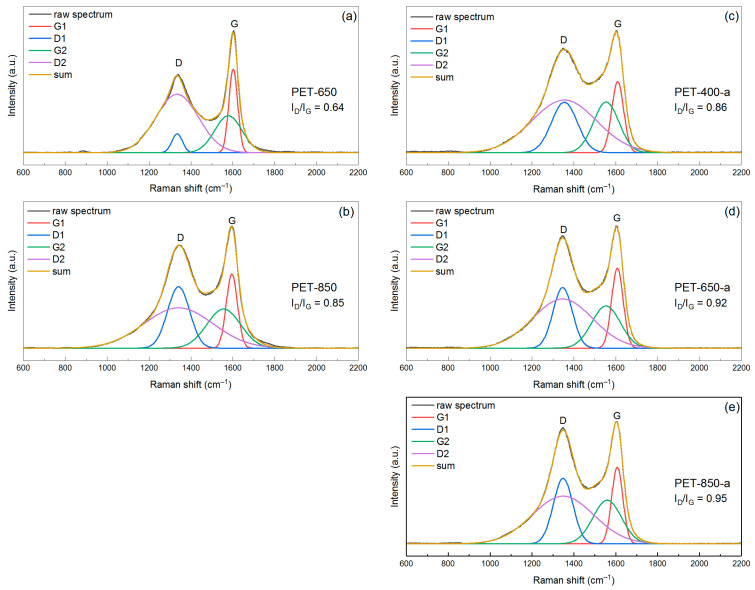
Raman spectra of the (**a**,**b**) pyrolyzed and (**c**–**e**) post-activated samples of PET-derived carbons.

In Figure 6, Raman spectra of pyrolyzed and post-activated PET-derived carbons are presented. The spectrum for the sample pyrolyzed at 400 °C is not available because this material produces a very noisy and uninformative spectrum due to fluorescence interference. Assessing the appearance and shape of the spectra of the pyrolyzed samples, as well as the values of the raw D- and G-band intensity ratios (which are 0.64 and 0.85 for PET-650 and PET-850, respectively), it can be stated that PET-650 is more ordered, has fewer defects and, as a consequence, should have larger crystallites than PET-850. By applying the aforementioned spectra deconvolution approach, we obtained the parameters of the four Gaussians (Table S1). When using Equation (4), we used the ratio (*A_D*1*_* + *A_D*2*_*)/*A_G*1*_* because it gives more accurate values of crystallite size close to the values obtained through Sherrer equation for XRD data [47]. It was found that Raman spectra analysis showed crystallite sizes of 5.2 and 3.8 nm for PET-650 and PET-850, respectively. While the values of crystallite size for PET-850 sample calculated through Equations (3) and (4) are close, the values for PET-650 sample are very different.

The post-activated samples showed an evolution of the ratio of raw D- and G-peak intensities. The *I_D_*/*I_G_* ratio values were 0.86, 0.92 and 0.95 for PET-400-a, PET-650-a and PET-850-a, respectively, indicating an increase in the defect fraction. This change correlates well with the CHN elemental analysis data, which shows an increase in oxygen content in both pyrolyzed and post-activated samples. However, the change in the saddle height between the peaks also draws attention. Calculation of crystallite size from the peak parameters from Raman spectra showed that the crystallites are 3.5, 4.3, and 3.9 nm for PET-400-a, PET-650-a and PET-850-a, respectively (Table 2). In contrast to the crystallite size calculated from XRD data, where a distinct dependence of the crystallite size on the pyrolysis temperature can be seen, the Raman spectra analysis does not provide such a direct dependence of the crystallite size. It can be seen that the maximum crystallite size of 4.3 nm occurs in the activated sample, which had a preliminary pyrolysis temperature of 650 °C. In this case, increasing the pretreatment temperature decreases the crystallite size to 3.9 nm.

Initially, it seems that the results of Raman spectra analysis contradict the data obtained from XRD patterns analysis. However, the data of all mentioned methods of research begin to correlate well with each other if we assume some heterogeneity of samples by depth. This means that we suggest that the obtained material has different structure and morphology on the surface and within the volume. Additionally, Raman spectroscopy appeared to be more sensitive to the defective carbon material compared to XRD. Thus, according to elemental CHN analysis, the oxygen content of the samples increases with pyrolysis temperature, and the D-band on Raman spectra increases its intensity, which affects the ratio of integrated intensities of D- and G-peaks. Thus, Raman spectra analysis demonstrates a decrease in the size of ordered domains in the carbon sample.

The pyrolyzed PET-650 sample, which has low crystallinity according to XRD, is of particular interest. However, the Raman spectrum of this sample stands out against the background of other spectra, indicating a relatively high ordering of the carbon crystal structure. Given the low content of oxygen groups in this sample, we hypothesize that the surface layers of the material have higher crystallinity compared to the entire volume of the material. At the same time, the concentration of oxygen groups may be lower on the surface compared to the whole volume. FTIR spectrum of the PET-650 sample (Figure 2), which declares the surface chemistry of the material, confirms this.

Considering the large porosity of the materials, all of them are suitable for investigation as adsorbents for the purification of aqueous media.

### 3.2. Adsorption Properties of Pyrolyzed and Post-Activated PET Samples

In order to show the adsorption properties of PET-derived activated carbons, we investigated the obtained samples as adsorbents for purification of aqueous solution from methylene blue dye. 

**Figure 7:**
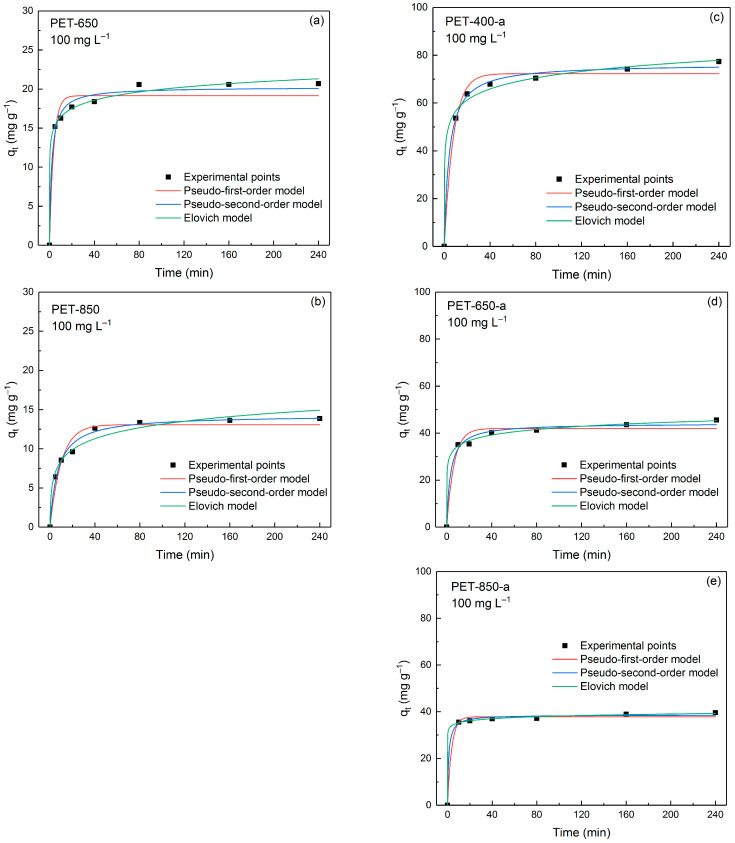
Effect of contact time on adsorption capacity of (**a**,**b**) pyrolyzed and (**c**–**e**) post-activated PET-derived carbons at 100 mg L^−1^ initial MB concentration.

The kinetics of MB adsorption was studied using a constant MB concentration of 100 mg L^−1^. In Figure 7, the adsorption behavior of MB onto PET-derived carbons is shown. As can be seen from the figure, most of the samples are characterized by rapid capture of dye molecules. Three kinetic models—namely pseudo-first-order (PFO), pseudo-second-order (PSO), and Elovich models—were applied to describe the experimental data.

Table 3 presents a comprehensive summary of key parameters derived from PFO, PSO and Elovich models, offering a detailed insight into the kinetics processes involved in the adsorption of MB onto the prepared carbon materials. The values of the constants and capacities of the samples were calculated using a non-linear regression method. The parameters obtained by PFO, PSO, and Elovich kinetic models are evaluated by coefficients of determination R^2^ and average relative error (ARE). Comparing the R^2^ and ARE values across the kinetic models employed, it is evident that the adsorption behavior is better described by the PSO and Elovich models. For example, for pyrolyzed PET-derived carbons the R^2^ value is not more than 0.9663 and the ARE values are approximately equal to 7%. Post-activated samples have a smaller error in PFO model fitting, but the R^2^ and ARE values are lower for PSO and Elovich models. The best-fitting models assume chemisorption associated with the interaction between oxygen groups on the surface of the carbon material and the cation of the dye molecule. Thus, PET-850 containing more oxygen groups according to CHN elemental analysis is described by PSO model better than PET-650.

In addition to the kinetic study, adsorption isotherms serve as valuable tools for analyzing the interaction between adsorbent and adsorbate. To describe the adsorption process of MB onto the obtained PET-derived carbon samples, the Langmuir and Freundlich models were employed. 

**Figure 8:**
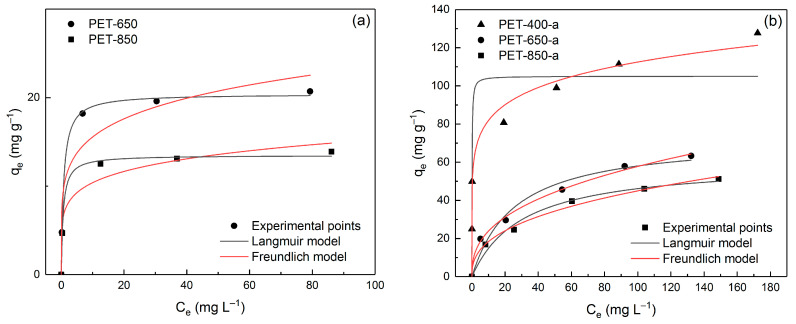
Langmuir and Freundlich model isotherms fitting data of MB adsorption onto prepared (**a**) pyrolyzed and (**b**) post-activated PET-derived carbons.

In Figure 8, the experimental data of dependence of adsorption values on MB concentration described by Langmuir and Freundlich models are presented.

It can be seen from the figure that the adsorption pattern is very different between pyrolyzed and post-activated samples. Table 4 demonstrates the outcomes of this isotherm analysis, presenting the calculated parameters using a non-linear regression technique.

The Langmuir model assesses monolayer adsorption on a homogenous surface, whereas the Freundlich model characterizes heterogeneous adsorption. The comparison of the obtained parameters unveils distinct adsorption behavior among the samples. As seen from Table 4, the R^2^ and ARE values indicate that adsorption onto pyrolyzed samples is better described by the Langmuir model, while the adsorption behavior of post-activated samples is better supported by the Freundlich model. This is explained by the fact that post-activation leads to etching (oxidation followed by carbon escape in the form of CO and CO_2_) of the carbon structure, including the expansion of already existing pores. The enlarged pores are more suitable for filling them with adsorbate in several layers. Although it is worth noting that the samples remain microporous for the most part. PET-400-a exhibits the highest maximum adsorption capacity *q_m_* of 105.1 mg g^−1^, emphasizing its superior capability to adsorb MB. PET-650-a and PET-850-a have lower *q_m_* values of 72.9 and 60.3 mg g^−1^, respectively. 

Although the adsorption characteristics of the obtained samples exhibit a strong correlation with the nitrogen adsorption/desorption data, a significant observation draws attention to a notable difference. Specifically, there are relatively low values of MB adsorption onto the pyrolyzed samples, despite their considerably high specific surface area. This discrepancy is particularly pronounced in the case of the PET-650 sample, which has a specific surface area of 504.2 m^2^ g^−1^ while displaying a modest MB adsorption value of only 20.4 mg g^−1^. A preceding investigation into the sample’s structural properties provides an explanation for this phenomenon. Our assumption is that the resulting carbon material is heterogeneous, featuring varying levels of oxygen groups on the surface. Intriguingly, post-activation does not lead to an increase in specific surface area; instead, contrary to expectations, the values surprisingly decrease (Table 1). However, when examining the removal of MB from aqueous solutions, more coherent results were obtained. We propose that the pyrolysis of PET leads to the creation of a rather porous carbon material. However, the challenge lies in the difficulty of MB molecules accessing these pores, primarily due to both a bottle-neck structure and the absence of oxygen groups.

The absence of oxygen groups compromises the hydrophilicity of the material, affecting its wettability and hindering the diffusion of the MB solution into the porous structure of the carbon material [48,49]. The data underscore that the PET-650 sample, containing minimal oxygen content, lacks sufficient hydrophilic properties, rendering it less effective as an adsorbent for aqueous solutions. Post-activation serves a dual purpose: firstly, it removes the amorphous/defective carbon layer, contributing significantly to the total specific surface area. Secondly, post-activation induces surface oxidation, enhancing the adsorption capacity of the carbon material for MB. This multifaceted insight highlights the interplay between surface characteristics and adsorption efficiency, offering a nuanced understanding of the material’s behavior in water purification applications.

To validate our hypothesis regarding the restricted pore accessibility attributed to the low number of oxide groups, we examined the adsorption behavior of the samples at different pH values. Under acidic conditions at pH 2.0, PET-400-a and PET-650 exhibited adsorption values of 49.8 and 19.8 mg g^−1^, respectively. These adsorption values are less than what these samples provided in neutral medium. In an alkaline environment with a pH of 12.0, these values increased up to 79.3 and 58.6 mg g^−1^ for the same samples. Notably, for the PET-650 sample, the adsorption value increased almost threefold. This supports our suggestion about the disadvantages of the sample obtained by direct pyrolysis (PET-650), which consists of the low content of oxygen groups on the surface, which reduces the availability of a relatively developed pore system in the carbon structure. The alkaline medium with higher pH increases the number of hydroxyl groups and as a consequence negatively charged sites on the adsorbent surface, which promotes the interaction between the cationic dye and the adsorbent.

Commonly, the mechanism of interaction between the carbon adsorbent and MB dye consists of electrostatic interactions between oxygen groups and the MB molecule as well as π- π interactions between the aromatic backbone of MB molecule and hexagonal rings of the carbon structure of the adsorbent [50]. The described studies on the structure of the PET-derived adsorbents indicate the same mechanisms.

**Figure 9:**
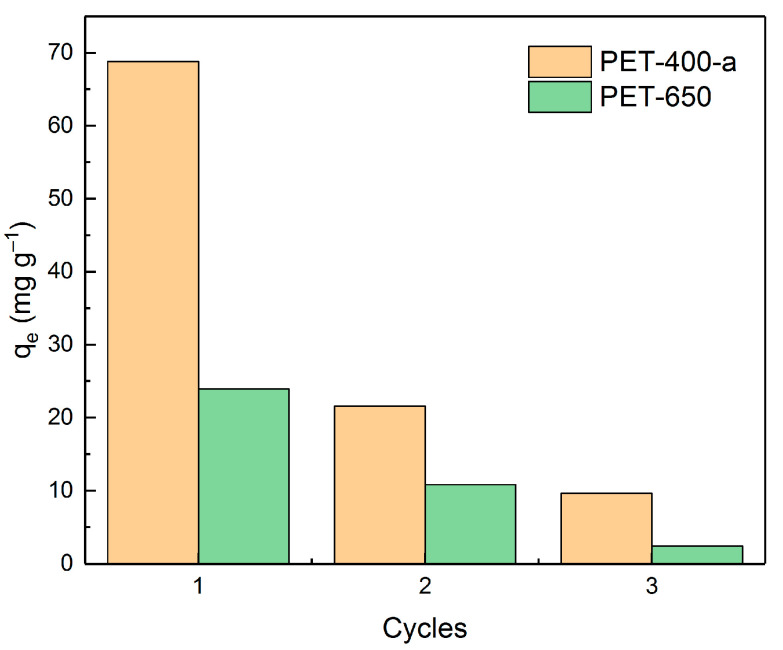
Dependence of adsorbent efficiency on the number of cycles of their application.

Further, the reusability of adsorbents was tested. For this purpose, the most efficient samples from each series (PET-400-a and PET-650) were selected and used in three cycles of MB adsorption (at MB concentration of 100 mg L^−1^ in a volume of 50 mL distilled water, adsorbent weight 0.05 g, equilibrium time 5 h) followed by desorption. Ethanol was used as an eluent. Figure 9 shows the adsorption capacity values of PET-400-a and PET-650 samples as a function of the amount of usage of these samples.

As can be seen from the dependence, after the first application of PET-400-a and PET-650, their efficiencies dramatically decrease down to 31% and 45%, respectively, of their initial adsorption values. This indicates a relatively low potential for their reuse. On the other hand, these materials can be considered as adsorbents that strongly bind harmful substances for their further neutralization or disposal.

Additionally, the PET-400-a sample was applied for the removal of MB from water from a real source. For this purpose, water from the Black Sea (Tuapse region, Russia) and the Moskva River (the sampling was taken in the center of the city, Moscow, Russia) was used, which was characterized by pH values equal to 8.2 and 8.3, respectively. These values correlate well with other studies [51]. The adsorption test conditions were the same as the previous ones at a dye concentration of 100 mg L^−1^. After adsorption for 5 h, the adsorption values for PET-400-a sample were 70.1 and 69.3 mg g^−1^ for seawater and river water, respectively. These values correspond well with the adsorption value obtained for PET-400-a sample by purification of bidistilled water. This confirms the efficiency of the obtained PET-derived carbon in the purification of a real water source.

### 3.3. Comparison with other Materials and their Adsorption Properties

Table 5 summarizes the synthesis conditions, specific surface area (SSA) and adsorption value of materials from other works investigating carbon adsorbents for MB water purification.

As can be seen from Table 5, the obtained carbon adsorbent is superior to other PET-derived carbons both in adsorption capacity and in time and energy costs for their production. Typically, furnaces with heating rates rarely exceeding 10 °C min^−1^ are used in the preparation of carbon materials. We note the work [54], which is devoted to the production of carbon from PET at a relatively low temperature of 500 °C. However, the adsorption value is less than many known adsorbents.

In the study [57], adsorbents based on multi-walled carbon nanotubes have been proposed. The adsorption value is slightly superior to the value obtained in our study. However, it is worth noting the incomparable cost of nanotubes compared to PET waste. In addition, the higher adsorption value was achieved due to additional functionalization of the nanotube surface with benzenesulfonate groups, which increases the cost of the adsorbent.

Activated carbons exhibit remarkable diversity, with some materials featuring surface areas exceeding 1000 m^2^ g^−1^. However, their production often entails significant time and energy investments. For instance, a method outlined in [58] suggests recycling old compact discs to yield porous carbon with a surface area of 1136.0 m^2^ g^−1^ and an adsorption capacity of 357.0 mg g^−1^. The process of achieving such high surface areas involves heating the material at 940 °C for 8 h with a heating rate of 1 °C min^−1^, which presents a notable drawback in terms of time and energy consumption.

Numerous studies focus on preparing porous adsorbents from biomass. For instance, in [59], the researchers produced a carbon material from Pequi almonds which had a surface area of 1923.0 m^2^ g^−1^ and an adsorption capacity of 500.0 mg g^−1^. Compared to PET waste, which is found worldwide, individual biomass sources are often geographically limited. Moreover, the utilization of biomass requires additional drying and purification stages.

Additionally, there are more complex techniques for fabricating carbon adsorbents, involving the combination of various carbon components. In such instances, the production process for each constituent tends to be notably complex and costly. For instance, in [60], an adsorbent featuring a surface area of 1361.9 m^2^ g^−1^ and an adsorption capacity of 174.8 mg g^−1^ is proposed. Although it surpasses our suggested adsorbent in performance, this method lacks economic viability and fails to address the challenge of PET waste utilization.

## 4. Conclusions

In summary, this study focused on the preparation of pyrolyzed and post-activated PET-derived carbons as a sustainable approach for converting polyethylene terephthalate waste. The investigation demonstrated their structural, porosity and adsorption properties, elucidating the impact of the synthesis temperature.

Notably, a comparative analysis revealed differing sensitivities of XRD and Raman spectroscopy methods towards these materials. The discrepancy in crystallite size calculations between XRD and Raman spectroscopy underscored the latter’s sensitivity to oxygen group content, introducing defects in the graphite layers of the carbon material and reducing crystallite sizes in the graphite-like phase. Additionally, porosity analysis via nitrogen adsorption/desorption indicated that post-activation led to a reduction in specific surface area for pyrolyzed samples. For instance, specific surface areas decreased from 504.2 to 439.0 m^2^ g^−1^ for PET-650 and from 221.0 to 166.0 m^2^ g^−1^ for PET-850 samples.

A novel finding of this study was the ability to directly obtain porous carbon materials through the pyrolysis of PET. Both pyrolyzed and post-activated carbons were evaluated as adsorbents for removing MB dye from aqueous solution. Despite a decrease in specific surface area after post-activation, adsorption tests demonstrated a positive effect attributed to changes in the surface chemistry of post-activated carbons, specifically the introduction of oxygen groups. PET-400-a sample (617.7 m^2^ g^−1^), derived from an incompletely degraded polymer, exhibited the highest MB adsorption value, reaching 127.7 mg g^−1^ at an MB solution concentration of 300 mg L^−1^. This not only highlights the efficiency of PET-derived carbons as adsorbents but also emphasizes the potential of pyrolysis as a direct and sustainable route to porous carbon materials with promising adsorption capabilities.

## Figures and Tables

**Table 1:** Porosity characteristics of the PET-derived carbon samples.

Sample	SSA, m^2^ g^−1^	V, cm^3^ g^−1^	C, wt%	O, wt%	H, wt%
PET-400	-	-	73.8	22.3	3.9
PET-550	521.5	0.257	93.0	3.3	3.7
PET-650	504.2	0.204	92.1	5.4	2.5
PET-750	414.1	0.197	87.7	11.3	1.0
PET-850	221.0	0.119	85.3	13.8	0.9
PET-400-a	617.7	0.289	89.3	10.3	0.4
PET-650-a	439.0	0.187	87.4	12.2	0.4
PET-850-a	166.0	0.074	87.0	12.7	0.3

**Table 2:** Crystallite size values calculated via XRD patterns and Raman spectra analysis.

Sample	^a^ D_XRD_, nm	^b^ D_Raman_, nm
PET-400	1.7	-
PET-450	1.7	-
PET-550	1.7	-
PET-650	1.7	5.2
PET-750	2.0	-
PET-800	2.2	-
PET-850	2.4	3.8
PET-400-a	2.6	3.5
PET-650-a	2.7	4.3
PET-850-a	2.9	4.0

^a^ Crystallite size values calculated via Sherrer equation (Equation (3)); ^b^ crystallite size values calculated via general equation (Equation (4)) from [39].

**Table 3:** Adsorption kinetic parameters for methylene blue adsorption (at concentration of 100 mg L^−1^) onto pyrolyzed and post-activated PET-derived carbons.

Parameters	PET-650	PET-850	PET-400-a	PET-650-a	PET-850-a
Pseudo-first-order					
q_1_ (mg g^−1^)	19.2	13.1	72.3	41.9	37.8
k_1_ (min^−1^)	0.2731	0.1002	0.1260	0.1549	0.2706
R^2^	0.9663	0.9637	0.9863	0.9664	0.9934
ARE (%)	7.00	6.96	3.18	4.52	2.06
Pseudo-second-order					
q_2_ (mg g^−1^)	20.3	14.3	76.4	44.2	38.6
k_2_ (g mg^−1^ min^−1^)	0.0246	0.0101	0.0031	0.0069	0.0245
R^2^	0.9895	0.9903	0.9972	0.9893	0.9969
ARE (%)	3.84	2.89	1.17	2.74	1.51
Elovich					
α (mg g^–1^ min^–1^)	6.86 × 10^3^	12.98	3.2 × 10^3^	8.38 × 10^3^	1.63
β (g mg^–1^)	0.6515	0.4911	0.1496	0.2934	0.7915
R^2^	0.9954	0.9726	0.9921	0.9952	0.9994
ARE (%)	1.78	5.03	1.83	1.3	0.53

**Table 4:** Langmuir and Freundlich isotherm model parameters for adsorption of MB onto pyrolyzed and post-activated PET samples.

Models	PET-650	PET-850	PET-400-a	PET-650-a	PET-850-a
Langmuir					
q_m_ (mg g^−1^)	20.4	13.5	105.1	72.9	60.3
K_L_ (L mg^−1^)	1.35	1.68	8.87	0.04	0.03
R^2^	0.9989	0.9972	0.8668	0.9708	0.9864
ARE (%)	1.85	2.39	15.24	16.57	10.72
Freundlich					
K_F_((mg g^−1^)(L mg^−1^)^1/n^)	10.31	7.1	59.6	9.9	7.5
n	5.59	6.02	7.25	2.60	2.57
R^2^	0.9268	0.9658	0.9400	0.9968	0.9937
ARE (%)	18.97	10.54	10.42	3.61	3.71

**Table 5:** Comparison of carbon material production conditions, its porosity and adsorption properties.

Carbonaceous Precursor	Synthesis Conditions	SSA (m^2^ g^−1^)	Adsorption Value (mg g^−1^)	Ref
PET	Post-activation 850 °C, 50 °C min^−1^, exposure time: 2 min, N_2_ atmosphere	617.7	127.7	This study
PET	800 °C, 8 °C min^−1^, exposure time: 90 min, autoclave	723.7	125	[52]
PET + waste ash	850 °C, 10 °C min^−1^, exposure time: 60 min, N_2_ atmosphere	485.0	92.3	[53]
PET	500 °C, 10 °C min^−1^, exposure time: 15 min, air atmosphere	378.8	43.9	[54]
PET	Post-activation 850 °C, exposure time: 25 min CO_2_ atmosphere	703.4	18.3	[55]
MWCNT	1050 °C, exposure time: 180 min, N_2_ atmosphere	537	62.5	[56]
MWCNT-S	-	233.0	150.2	[57]
Compact disc	Post-activation 940 °C, 1 °C min^−1^, exposure time: 480 min CO_2_ atmosphere	1136.0	357.0	[58]
Pequi almonds	Post-activation 800 °C, 5 °C min^−1^,	1923.0	500.0	[59]
GO-CNT/AC	Complex preparation (see [60])	1361.9	174.8	[60]

## Data Availability

Data are contained within the article and Supplementary Materials.

## Supplementary Materials

The following supporting information can be downloaded at: https://www.mdpi.com/article/10.3390/polym16070891/s1. Figure S1. Images of (a) the initial sliced PET sample; (b) PET pyrolyzed at 400 °C and (c) PET pyrolyzed at 400 °C and ground for further impregnation with the activating agent solution. Table S1. Calculated fitting parameters of the synthetic Raman peaks.
