# Supplementary material for: Application of Infrared Pyrolysis and Chemical Post-Activation in the Conversion of Polyethylene Terephthalate Waste into Porous Carbons for Water Purification
Source: Polymers (Basel). 2024 Mar 24;16(7):891. doi: 10.3390/polym16070891 (PMC11013903; doi:10.3390/polym16070891)
Supplement: Supplementary file 1 [file polymers-16-00891-s001.zip › polymers-2912381-supplementary.pdf]

**Supplementary material**

# Application of infrared pyrolysis and chemical post-activation in the conversion of polyethylene terephthalate waste into porous carbons for water purification

M.N. Efimov[a,*], A.A. Vasilev[a,b*], D.G. Muratov[b], A.A. Panin[a], M.S. Malozovskaya[c], G.P. Karpacheva[a]

[a] *A.V. Topchiev Institute of Petrochemical Synthesis RAS, Leninskiy Prospekt 29, 119991 Moscow, Russia*

[b] *National University of Science and Technology "MISiS", Leninskiy Prospekt 4, 119049 Moscow, Russia*

[c] *National Research Center "Kurchatov Institute", Academic Kurchatov Sq. 1, 123182 Moscow, Russia*

[*]Corresponding author: M.N. Efimov. Email: efimov@ips.ac.ru. A.A. Vasilev. Email: raver.vasiljev@mail.ru

## 1. Adsorption isotherms

Three equilibrium models, namely Langmuir, Freundlich and Sips were used to fit experimental data. Langmuir model describes the monolayer homogeneous distribution of the adsorbate molecules according to Equation (S1).

$$q_e = \frac{q_m K_L C_e}{1 + K_L C_e} \quad \text{(S1)}$$

where $C_e$ is the equilibrium concentration of MB (mg $L^{-1}$); $q_e$ is the amount adsorbed at the equilibrium (mg $g^{-1}$); $K_L$ is the Langmuir constant related to the energy of adsorption; $q_m$ is the maximum adsorption capacity (mg $g^{-1}$).

Freundlich adsorption model assumes heterogeneous surface energies and multilayer distribution of the adsorbate. This model is described by Equation (S2).

$$q_e = K_F C_e^{1/n} \quad \text{(S2)}$$

where $q_e$ is the amount adsorbed at the equilibrium (mg $g^{-1}$); $K_F$ ((mg $g^{-1}$)(L $mg^{-1}$)$^{1/n}$) and $n$ are the Freundlich constants; $C_e$ is the equilibrium adsorbate concentration (mg $L^{-1}$).

**2. Kinetics equations**

The PFO and PSO models are described by Equation (S4) and (S5).

$$q_t = q_1(1 - \exp(-k_1 t)) \quad \text{(S4)}$$

$$\frac{t}{q_t} = \frac{1}{k_2 q_2^2} + \frac{t}{q_2} \quad \text{(S5)}$$

where $q_1$, $q_2$ and $q_t$ are the theoretical and time adsorption capacity (mg $g^{-1}$), respectively; $k_1$ and $k_2$ are pseudo-first-order rate constant ($min^{-1}$) and pseudo-second-order rate constant (g $mg^{-1}$ $min^{-1}$).

The Elovich equation is expressed as:

$$q_t = \frac{1}{\beta}\ln(1 + \alpha\beta t) \quad \text{(S6)}$$

where $q_t$ is the time adsorption capacity (mg $g^{-1}$); α is the initial sorption rate (mg $g^{-1}$ $min^{-1}$) and β is the desorption constant (g $mg^{-1}$); $t$ is the contact time (min).

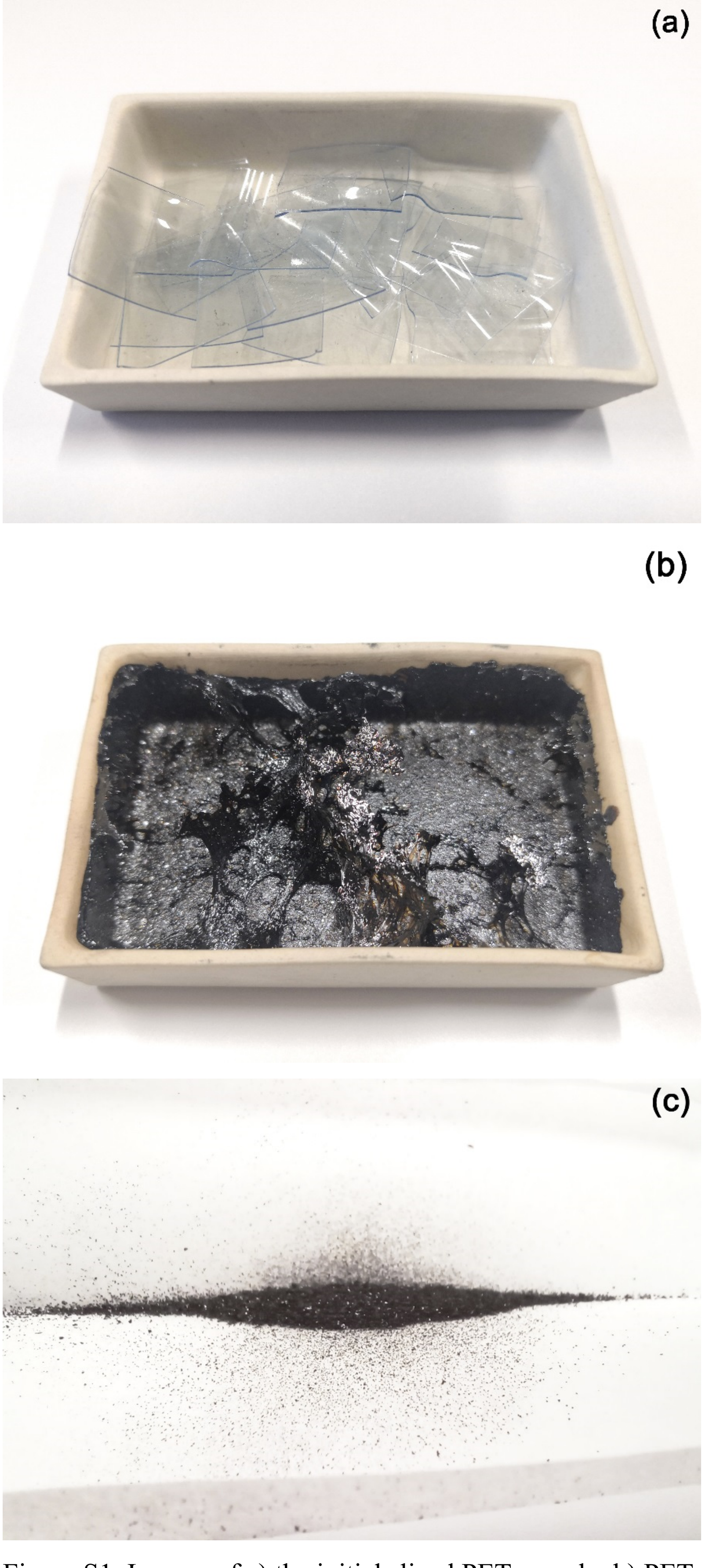


Figure S1. Images of a) the initial sliced PET sample; b) PET pyrolyzed at 400 °C and c) PET pyrolyzed at 400 °C and ground for further impregnation with the activating agent solution.

Table S1. Calculated fitting parameters of the synthetic Raman peaks.

| Sample | Peak | Center | Height | Area | FWHM | $I_{D1}/I_{G1}$ | $A_{(D1+D2)}/A_{G1}$ | D, nm |
|---|---|---|---|---|---|---|---|---|
| PET-650 | G1 | 1601.93 | 0.68292 | 36.0675 | 49.6151 | 0.23 | 3.68 | 5.23 |
| | D1 | 1334.21 | 0.154869 | 9.85776 | 59.7972 | | | |
| | G2 | 1578.81 | 0.302937 | 49.5173 | 153.558 | | | |
| | D2 | 1334.21 | 0.478426 | 122.821 | 241.17 | | | |
| PET-850 | G1 | 1595.47 | 0.607395 | 40.5585 | 62.7305 | 0.83 | 5.03 | 3.82 |
| | D1 | 1341 | 0.50408 | 66.82 | 124.53 | | | |
| | G2 | 1556.72 | 0.320724 | 65.1946 | 190.962 | | | |
| | D2 | 1341 | 0.33024 | 137.165 | 390.194 | | | |
| PET-400-a | G1 | 1609.09 | 0.582671 | 41.668 | 67.1811 | 0.71 | 5.56 | 3.46 |
| | D1 | 1354.59 | 0.413991 | 63.9633 | 145.147 | | | |
| | G2 | 1554.35 | 0.415209 | 63.4551 | 143.571 | | | |
| | D2 | 1354.59 | 0.43128 | 167.883 | 365.691 | | | |
| PET-650-a | G1 | 1607.6 | 0.654698 | 45.4442 | 65.2086 | 0.76 | 4.46 | 4.31 |
| | D1 | 1346.4 | 0.496292 | 57.5873 | 109.008 | | | |
| | G2 | 1553.57 | 0.345772 | 59.5015 | 161.661 | | | |
| | D2 | 1346.4 | 0.402865 | 145.078 | 338.305 | | | |
| PET-850-a | G1 | 1607 | 0.627664 | 42.2114 | 63.1786 | 0.85 | 4.91 | 3.92 |
| | D1 | 1348.5 | 0.536505 | 62.7798 | 109.929 | | | |
| | G2 | 1559.18 | 0.357631 | 61.2072 | 160.781 | | | |
| | D2 | 1348.5 | 0.390356 | 144.432 | 347.592 | | | |
